# Effectiveness and safety of Xinkeshu on coronary artery disease patients combined with anxiety and depression symptoms after percutaneous coronary intervention: A protocol for systematic review and meta-analysis

**DOI:** 10.1097/MD.0000000000027912

**Published:** 2021-11-19

**Authors:** Mingtai Chen, Guofu Zhong, Ling Men, Qiang Liu, Jienan Luan

**Affiliations:** a Department of Cardiovascular Disease, Shenzhen Traditional Chinese Medicine Hospital, Shenzhen, China; b Intensive Care Unit, Shenzhen Traditional Chinese Medicine Hospital, Guangzhou University of Chinese Medicine, Shenzhen, China; c Nephrology Department, Shenzhen Traditional Chinese Medicine Hospital, Shenzhen, China.

**Keywords:** anxiety and depression, meta-analysis, percutaneous coronary intervention, protocol, randomized trial, Xinkeshu

## Abstract

**Background:**

It's known that coronary heart disease (CHD) patients after percutaneous coronary intervention (PCI) was significantly associated with anxiety and depression symptoms. Several studies have showed that Xinkeshu tablet (XKS), a kind of Chinese herbal medicine, could effectively improve post-PCI postoperative mood disorders in CHD patients. However, the intensity of evidence has been poor, limiting the further clinical application of XKS to patients above. This systematic review and meta-analysis will assess the effectiveness and safety of studies of XKS in CHD patients with anxiety and depression symptoms after PCI.

**Methods:**

A systematic literature search for articles up to December 2021 will be performed in following electronic databases: PubMed, Embase, the Cochrane Library, China National Knowledge Infrastructure, Chinese Scientific Journals Database Database, Chinese Biomedical Database, Chinese Biomedical Literature Service System, and Wanfang Database. Inclusion criteria are randomized controlled trials of XKS applied on patients with CHD and depression. The primary outcome measures will be CHD-related clinical evaluation (frequency of acute attack angina, severity of angina pectoris, electrocardiographic changes, amount of nitroglycerin) and the scores or reducing fractions of depressive and anxiety measuring scales (the Hospital Anxiety/Depression Scale or other widely used anxiety/depression scale). The safety outcome measures will be adverse events, liver and kidney function. RevMan 5.3 software will be used for data synthesis, sensitivity analysis, subgroup analysis, and risk of bias assessment. A funnel plot will be developed to evaluate reporting bias. Stata 12.0 will be used for meta-regression and Egger tests. We will use the Grading of Recommendations Assessment, Development and Evaluation system to assess the quality of evidence.

**Discussion:**

This study will provide a high-quality synthesis of the effects and safety of XKS for CHD patients with anxiety and depression symptoms after PCI.

**Ethics and dissemination:**

This systematic review does not require ethics approval and will be submitted to a peer-reviewed journal.

Trial registration number PROSPERO CRD42019131346.

## 1 Introduction

Anxiety and depression symptoms are predictors of a worse prognosis in coronary heart disease (CHD) patients.^[1–4]^ It was confirmed that CHD patients after percutaneous coronary intervention (PCI) were significantly associated with anxiety and depression symptoms at different follow-up time points.^[5]^ In addition, anxiety and depression symptoms of CHD patients aggravated significantly after PCI.^[6]^ In the population of CHD patients after PCI, anxiety and depression symptoms not only were associated with a increased risk for major adverse cardiac events but were shown to severely disrupt long-term quality of life.^[4–7]^ As far as the severity of this issue was concerned, several therapeutic measures were tried to solve this problem, such as anti-anxiety or depression therapy, Tai Chi, cardiac rehabilitation (CR), etc.^[8–10]^ However, the results were dissatisfying, in which the side effects of anti-anxiety depression therapy limited their use overall in the long-term treatment of CHD patients and CR couldn’t significantly improve anxiety/depression of CHD patients.^[11,12]^

Chinese herbal medicine has been used clinically as therapy treatment for thousands of years. Xinkeshu tablet (XKS) as a complex matrix comprises 5 medicinal materials or extracts thereof, including Salvia miltiorrhiza Bge, Radix Puerariae, Hawthorn, Panax Notoginseng, and Radix Aucklandiae. XKS, which was proven anti-anxiety effects and associated with little toxicity, was widely applied as an alternative therapy for the CHD combined with anxiety and depression in China. Several studies showed that XKS could effectively improve post-PCI postoperative mood disorders in patients with CHD and improve the quality of life.^[13–17]^

Although numerous clinical studies and reviews have assessed XKS in the treatment of CHD patients with anxiety and depression, systematic review to evaluate the effectiveness and safety of XKS in such patient population was rare. We identified only one similar meta-analysis evaluating the effectiveness of XKS therapies in CHD patients with anxiety and depression, though the study had certain limitations.^[18]^ There were 18 randomized clinical trials (RCTs) included in the meta-analysis by Yuan et al.^[18]^ However, the primary outcome measures of the CHD-related clinical evaluations and anxiety/depression score scale were relatively insufficient; and the subgroup analysis about whether the patients experienced PCI treatment were not conducted. In view of the shortcomings of previous studies and the incomplete evidence regarding the widespread use of XKS, this meta-analysis aimed to summarize the effectiveness and safety of XKS in treating CHD patients with anxiety and depression after PCI.

## 2 Methods and analysis

### 2.1 Registration

The study protocol has been registered in the international prospective register of systematic review (PROSPERO). The trial registration number of PROSPERO is CRD42019131346. The procedure of this protocol will be conducted according to the Preferred Reporting Item for Systematic Review and Meta-analysis Protocols guidelines.^[19]^

### 2.2 Eligibility criteria

#### 2.2.1 Type of study

*Inclusion:* We will include all the RCTs that investigated the effectiveness and safety of XKS combined with conventional pharmacotherapy for the treatment of CHD patients with anxiety and depression after PCI.

*Exclusion:* The studies will be excluded if it is not an RCT (namely, observational cohort and case–control studies, case reports, experimental studies, and reviews).

#### 2.2.2 Participants

*Inclusion:* The study will include adult (18–85 years) CHD patients with anxiety and depression after PCI regardless of sex, ethnicity, education, or economic status and whether or not they were out- or in-patients. The diagnostic criteria for CHD, anxiety and depression will be as follows.

1. The diagnostic criteria of CHD should be confirmed according to one of the past or current definitions: Report of the Joint International Society and Federation of Cardiology/World Health Organization task force on standardization of clinical nomenclature of ischaemic heart disease, or the American College of Cardiology/American Heart Association guideline update for the management of patients with chronic stable angina or Chinese Association of Cardiology or unstable angina pectoris diagnosis and treatment recommendations.^[20–22]^
2. Anxiety and depression must be defined as anxiety disorder or clinical anxiety and depressive disorder or clinical depression diagnosed according to the Diagnostic and Statistical Manual of Mental Disorders, the International Classification of Diseases by a standardized interview (e.g., Structured Clinical Interview, Composite International Diagnostic Interview), or the Chinese Classification of Mental Disorders.^[23–25]^

*Exclusion:* Patients with either CHD or depression or anxiety only will be excluded. Patients with severe respiratory disease, acute infectious disease, severe heart disease, severe liver disease, or tumors will be excluded.

### 2.3 Interventions

*Inclusion:* Eligible interventions will be those involving a combination of XKS and conventional pharmacotherapy. The same conventional pharmacotherapy must be used in the control group.

*Exclusion:* Trials that include other co-interventions such as another herbal formula, acupuncture, cupping, moxibustion, massage, yoga, qigong, Tai Chi, or aromatherapy will be excluded.

### 2.4 Outcome

*Inclusion:* The primary outcome measures will include the following: CHD-related clinical evaluation (frequency of acute angina, severity of angina pectoris, electrocardiographic changes, dose of nitroglycerin), the scores or reduction in scales measuring depression and anxiety (i.e., the Hospital Anxiety and Depression Scale or other widely used anxiety/depression scale). The secondary outcome measures will include the following: total cholesterol, triglyceride, low-density lipoprotein cholesterol and high-density lipoprotein cholesterol levels, and the Traditional Chinese Medicine (TCM) syndrome scale. The safety outcomes will include the following: adverse events (such as digestive symptoms, headache, dizziness, skin rash etc), liver or kidney toxicity measured by serum markers.

*Exclusion:* The outcome measures not requested in this study will be excluded.

### 2.5 Search strategy

The following electronic bibliographic databases will be searched from inception to July 2020: PubMed, Embase, the Cochrane Library, China National Knowledge Infrastructure, Chinese Scientific Journals Database Database, Chinese Biomedical Database, Chinese Biomedical Literature Service System and Wanfang Database. A manual search of key journals and of the reference lists of reviews captured by the initial searches will also be performed. There will be no limits on the language of publication. Only clinical trials will be included and searched. The following sources will also be searched to identify clinical trials that are in progress or completed: Clinical Trials.gov and WHO clinical trials registry. Any additional relevant studies will also be retrieved from the reference lists of systematic reviews and included studies. If possible, we will map search terms to controlled vocabulary. In addition, the search strategy for selecting the fields of title, abstract or keyword will differ depending on the characteristics of the databases. Search terms will be grouped into three blocks (see Table 1).

**Table 1:** Search items.

Search block	Search items
Participants	Cardiac failure OR heart decompensation OR heart failure OR right-sided heart failure OR myocardial failure OR congestive heart failure OR left sided heart failure OR preserved ejection fraction OR preserved ejection fraction heart failure OR heart failure, preserved ejection fraction OR diastolic heart failure OR heart failure with preserved left ventricular ejection fraction OR HFpEF
Intervention	Baduanjin exercise OR Baduanjin OR BDJ OR BDJE OR Qigong OR eight section brocades OR regimen OR Chinese regimen OR Chinese ancient regimen OR rehabilitation exercise OR Medicine, Chinese Traditional OR Traditional Chinese Medicine OR Chung I Hsueh OR Hsueh, Chung I OR Traditional Medicine, Chinese OR Zhong Yi Xue OR Chinese Traditional Medicine OR Chinese Medicine, Traditional
Study design	Randomized controlled trial OR controlled clinical trial OR randomized OR placebo OR drug therapy OR randomly OR trial OR groups

### 2.6 Study selection and data extraction

**Figure 1:**
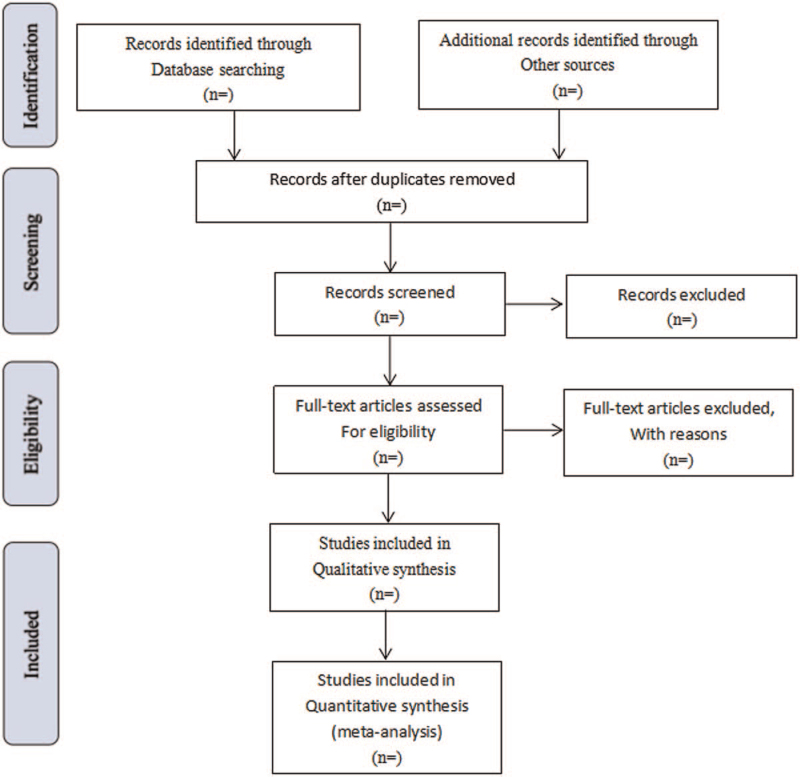
Flow diagram of study selection process. PubMed, Embase, the Cochrane Library, CNKI, VIP Database, CBM, SinoMed, and Wanfang Database.

Literature retrieved citations will be managed by EndNote X7 software. Two authors (CM and ZG) will independently screen the titles and abstracts of all the studies retrieved in the above electronic databases to identify potentially eligible studies. Articles that are duplicated or have not met the eligibility criteria, interventions and outcomes in this study will be excluded. After filtering the final eligible articles, the data from the included articles will be extracted independently by 2 authors (CM and ML). Disagreements will be resolved by discussion or arbitrated by a third author if needed. The following categories of data will be extracted: first author, publication year, diagnose information, age, sex, trial characteristics, interventions and controls, participants, study methodology, outcomes, and adverse events (see Fig. 1).

### 2.7 Risk of bias assessment

The methodological quality of the eligible studies will be evaluated according to the Cochrane Collaboration's tool for assessing risk of bias. The assessment details include: sequence generation, allocation concealment, blinding of participants and personnel, blinding of outcome assessors, incomplete outcome data, selective reporting, and other sources of bias. Each domain will be assessed as “low risk,” “high risk,” or “unclear risk” according to the description details of eligible studies.

### 2.8 Data synthesis and statistical analysis

Statistical analyses will be conducted with RevMan 5.3 software provided by Cochrane Collaboration (London, United Kingdom). The overall effect sizes will be determined as the mean difference for continuous outcomes, the odds ratio for dichotomous outcomes with their 95% credible intervals. The *Q* and *I*^2^ test statistics will be calculated to determine the amount of heterogeneity. For the *Q* statistic, *P* < .05 will be considered to indicate significant differences. For the *I*^2^ statistic, *I*^2^ < 25% indicates no significant heterogeneity, *I*^2^ = 25–50% is considered moderate heterogeneity and *I*^2^ > 50% indicates strong heterogeneity. We will use fixed effects models if there is no heterogeneity among studies, and random effects models if there is heterogeneity.

### 2.9 Sensitivity analysis, subgroup analysis, and meta-regression

If the heterogeneity or inconsistency among the studies is detected, a sensitivity analysis or subgroup analysis or meta-regression (conducted by Stata 12.0) analysis will be performed. Subgroup analysis will be conducted to explore potential sources of heterogeneity according to the characteristics of studies, including sample size, types of CHD, severity of depression, severity of anxiety, dose of XKS, treatment duration and other relevant parameters. If data extraction is insufficient, we will create a qualitative synthesis.

### 2.10 Publication bias

A funnel plot will be developed to evaluate reporting bias of the included studies. We will use Egger tests (conducted by Stata 12.0) to assess funnel plot symmetry and will interpret values of *P* < .1 as statistically significant.

### 2.11 Quality of evidence

We will also assess the quality of evidence for the main outcomes with the Grading of Recommendations Assessment, Development and Evaluation approach. Five items will be investigated, including limitations in study design, inconsistency, inaccuracies, indirectness, and publication bias.

### 2.12 Patient and public involvement

The patients and/or public will not be involved because this study uses secondary sources for analysis.

## 3 Discussion

We plan to conduct this meta-analysis to assess the effectiveness and safety of XKS for CHD patients with anxiety and depression after PCI. However, there may be some limitations because this is a retrospective meta-analysis. First, during the search, there is the inevitable potential that unpublished studies will not be identified which will introduce some bias. Besides, some grey literature may be difficult to retrieve, possibly leading to a selection bias in the literature. Moreover, some secondary outcome measures may not be completely reported. However, we expect that the results of this study will be able to propose clinical recommendations for CHD patients with anxiety and depression after PCI in clinical practices and provide more objective and reliable evidence supporting use of the XKS.

## Author contributions

Qiang Liu and Mingtai Chen conceived the study and drafted the protocol. Jienan Luan and Qiang Liu revised it. Mingtai Chen, Guofu Zhong, and Ling Men developed the search strategies, will conduct data collection and analyse the data independently. All authors will approve the final.

**Conceptualization:** Mingtai Chen, Qiang Liu, Jienan Luan.

**Data curation:** Mingtai Chen, Guofu Zhong.

**Formal analysis:** Mingtai Chen, Guofu Zhong.

**Funding acquisition:** Qiang Liu, Jienan Luan.

**Investigation:** Mingtai Chen, Ling Men.

**Methodology:** Mingtai Chen, Guofu Zhong, Ling Men.

**Project administration:** Mingtai Chen, Jienan Luan.

**Resources:** Ling Men.

**Supervision:** Jienan Luan.

**Writing – original draft:** Mingtai Chen.

**Writing – review & editing:** Ling Men, Qiang Liu, Jienan Luan.
